# Synergistic treatment strategy: combining CAR-NK cell therapy and radiotherapy to combat solid tumors

**DOI:** 10.3389/fimmu.2023.1298683

**Published:** 2023-12-15

**Authors:** Jie He, Yushan Yan, Jun Zhang, Zhiming Wei, Huashun Li, Ligang Xing

**Affiliations:** ^1^ Department of Oncology, The Affiliated Hospital of Southwest Medical University, Luzhou, China; ^2^ Shandong Cancer Hospital and Institute, Shandong First Medical University and Shandong Academy of Medical Sciences, Jinan, China; ^3^ Asclepius (Soochow) Technology Company Group, Suzhou, Jiangsu, China

**Keywords:** chimeric antigen receptor, radiotherapy, solid tumors, tumor microenvironment, natural killer cells

## Abstract

Immunotherapy, notably chimeric antigen receptor (CAR) modified natural killer (NK) cell therapy, has shown exciting promise in the treatment of hematologic malignancies due to its unique advantages including fewer side effects, diverse activation mechanisms, and wide availability. However, CAR-NK cell therapies have demonstrated limited efficacy against solid tumors, primarily due to challenges posed by the solid tumor microenvironment. In contrast, radiotherapy, a well-established treatment modality, has been proven to modulate the tumor microenvironment and facilitate immune cell infiltration. With these observations, we hypothesize that a novel therapeutic strategy integrating CAR-NK cell therapy with radiotherapy could enhance the ability to treat solid tumors. This hypothesis aims to address the obstacles CAR-NK cell therapies face within the solid tumor microenvironment and explore the potential efficacy of their combination with radiotherapy. By capitalizing on the synergistic advantages of CAR-NK cell therapy and radiotherapy, we posit that this could lead to improved prognoses for patients with solid tumors.

## 1 Introduction

With the rapid development of immunotherapy in recent years, people have refocused on the impact of the immune system on tumors. Adoptive cell therapy (ACT) is an important treatment approach. The previous ACT involved collecting immune cells from the patient’s body, culturing and expanding them *in vitro*, and reinjecting them into the body. However, it remains controversial due to its lack of specificity and limited efficacy. With the advancement of genetic engineering technology, researchers have developed an immune cell modified with the chimeric antigen receptor (CAR). This technology involves adding a specific antigen-recognition receptor on the surface of immune cells, which can recognize tumor-specific proteins and greatly enhance the targeting ability of ACT. The classical CAR mainly consists of an antigen-binding domain, a hinge domain, a transmembrane domain, and an intracellular signaling domain. Among them, the antigen-binding domain is composed of the variable region of heavy (VH) and light (VL) chains connected via a flexible linker to form a single-chain fragment variable (scFv) in the extracellular region When the CAR recognizes and binds to the antigen, its intracellular signaling domain sends a signal to activate downstream pathways and stimulate the immune cells to exert their effector functions (1, 2).

The most widely studied approach is CAR-modified T (CAR-T) cell therapy, which has exhibited remarkable efficacy in hematologic malignancies (3) and has been approved by regulatory authorities for treating such tumors. However, it has some drawbacks, including inducing severe cytokine release syndrome and neurotoxicity, a long and expensive production process, and limited therapeutic efficacy on solid tumors (4). As a result, researchers have shifted their attention to NK cells and developed CAR-NK cell therapy as a potential alternative. CAR-NK cells present several advantages over CAR-T cells, such as a lower risk of cytokine release syndrome, absence of graft-versus-host disease (GvHD), and multiple mechanisms of inducing cytotoxicity (5–7). Additionally, NK cells can be extracted from peripheral blood, cord blood, induced pluripotent stem cells, and NK cell lines. In a recent phase I/II clinical trial targeting CD19-positive B-cell lymphoma, CD19-CAR-NK cells exhibited potent anti-tumor effects (8). CAR-NK cells have been observed to have promising therapeutic effects on solid tumors in preclinical studies (9, 10), and corresponding clinical trials are currently underway, but their results have not yet been published. Nevertheless, for an effective CAR-NK cell therapy, it remains a critical challenge to overcome the immunosuppressive property of the solid tumor microenvironment (TME) that hinders the activity and function of NK cells.

Radiation therapy, as a conventional tumor treatment, has recently garnered attention due to its immunomodulatory effects. Increasing evidence suggests that it can reshape the TME and enhance immune cell infiltration (11–13). Several preclinical studies have investigated the combination of CAR-T cell therapy and radiotherapy for treating solid tumors and yielded promising outcomes (**Table 1**). NK cells, as a component of the innate immune system, possess distinctive activation mechanisms. Their combination with radiotherapy may alter the balance between their activation and inhibition signals, thereby augmenting the NK cell function. The combination of radiation therapy with CAR-NK cell therapy may represent a promising strategy for the treatment of solid tumors. In this review, we will focus on the mechanisms and challenges of this combination therapy.

**Table 1:** Preclinical trials of CAR-T cell therapy combined with radiotherapy.

Tumor type	RT dose	Result	Reference
Breast cancer	10 Gy	Radiotherapy enhanced the infiltration and cytotoxicity of CAR-T cells by activating the NF-κB/ICAM-1 signaling pathway.	Zhou et al. (14)
Glioblastoma	4 Gy	Radiotherapy increased the cytotoxicity of NKG2D-CAR-T cells against glioblastoma cells.	Weiss et al. (15)
Glioblastoma	5 Gy	Radiotherapy enhanced the infiltration of CAR-T cells into tumors, and combination therapy prolonged the survival time of the model mice of glioblastoma.	Murty et al. (16)
Pancreatic cancer	2 Gy	Radiotherapy increased the sensitivity of tumor cells to TRAIL-mediated apoptosis and enhanced the cytotoxicity of CAR-T cells.	DeSelm et al. (17)

CAR-T, chimeric antigen receptor-modified T; RT, radiotherapy; NF-κB, nuclear factor κB; ICAM-1, intercellular adhesion molecule 1; NKG2D, natural killer group 2 member D; TRAIL, TNF-related apoptosis-inducing ligand.

## 2 Mechanisms of CAR-NK cell recognizing and killing cancer cells

### 2.1 Intrinsic mechanism

**Figure 1:**
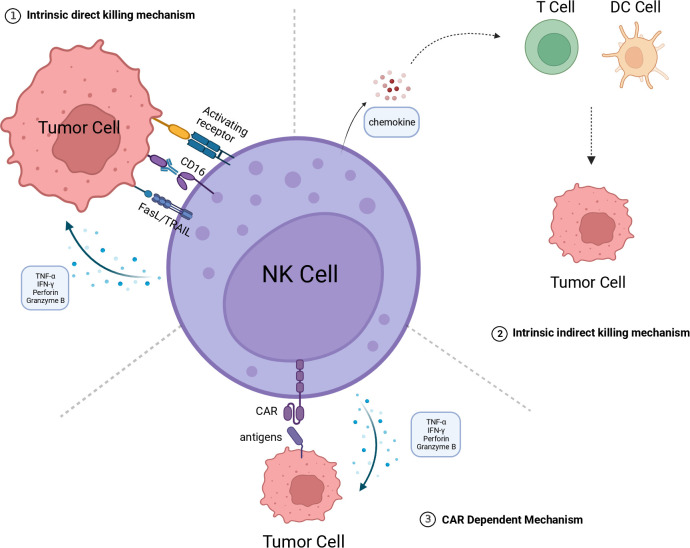
The killing mechanism of CAR-NK. 1.Intrinsic direct killing mechanism: NK cells directly kill tumor cells through the following mechanisms: Releasing perforin and granzyme B to directly induce tumor cell lysis; Secreting cytokines TNF-α and IFN-γ to induce apoptosis in tumor cells; Mediating tumor cell apoptosis through the FasL/TRAIL pathway; Exerting antibody-dependent cellular cytotoxicity (ADCC) mediated by CD16. 2. Intrinsic indirect killing mechanism NK cells have the ability to secrete chemokines, recruiting T cells, DC cells, and other immune cells to engage in collaborative cytotoxicity against tumor cells. 3. CAR dependent mechanism The CAR structure can recognize tumor-specific antigens, thereby activating NK cells to kill tumor cells by releasing perforin and granzyme B, among others. The figure is created with BioRender.com. CAR, chimeric antigen receptor; DC, dendritic cell; NK, natural killer; FasL, Fas ligand; TRAIL, TNF-related apoptosis-inducing ligand; TNF-α, tumor necrosis factor alpha; IFN-γ, interferon-gamma.

NK cells are a subset of innate immune cells that lack specific antigen-recognition receptors. They can rapidly recognize and eliminate tumor cells and infected cells without the need for prior sensitization to antigens (18). The activation and regulation of NK cells are determined by a delicate balance between activation and inhibition signals integrated through their surface receptors. Under normal physiological conditions, the inhibitory receptors on NK cells bind to Major Histocompatibility Complex class I molecules (MHC-I) on normal cells to transmit inhibitory signals and put NK cells in an inactive state, thereby preventing normal cells from being killed. However, during tumorigenesis or infection, the downregulation of MHC-I expression in tumor and infected cells disrupts the inhibitory signals, leading to NK cells being activated through a recognition mechanism called “missing-self” (19). Moreover, there are multiple activating receptors on the surface of NK cells, enabling them to recognize and bind to overexpressed or aberrantly expressed proteins on the surface of tumor or infected cells, thereby triggering NK cell activation. The activated NK cells employ diverse mechanisms to directly eliminate target cells, including the release of perforin and granzyme. Moreover, they can induce cell apoptosis through the Fas Ligand/TNF-Related Apoptosis-Inducing Ligand (FasL/TRAIL) signaling pathway or by secreting IFN-γ and TNF-α to engage in receptor-mediated interactions with target cells. Additionally, the Fc receptor CD16 on the surface of NK cells can bind to the Fc region of antibodies and trigger antibody-dependent cell-mediated cytotoxicity (ADCC) to clear target cells (20, 21) (**Figure 1**).

### 2.2 CAR-dependent mechanism

During tumor development, tumor cells often downregulate related surface antigens or upregulate some inhibitory receptors to evade immune surveillance, making it difficult for immune cells to recognize the tumor (22). Meanwhile, specific antigens are expressed in certain tumors. These specific antigens can serve as targets for tumor therapy. Installing a chimeric antigen receptor (CAR) structure on immune cells can make them recognize specific antigens. When the CAR binds to the target antigen, the activation signal is transduced into the cell to stimulate NK cell activation and release cytotoxic substances (23) (**Figure 1**). CAR structures have currently been developed to the fourth generation. The first-generation molecule consists of a synthetic extracellular receptor for antigen recognition, a transmembrane domain, and an intracellular signaling domain. The second and third-generation CAR molecules have one or more intracellular co-stimulatory domains to enhance signal transduction. The fourth-generation CAR, also known as armored CAR, includes molecular payloads that endow CAR-NK cells with additional characteristics and functions. Most CARs used for CAR-NK studies have the same structures as those employed for CAR-T cell therapy because they share the same signal pathways and co-stimulatory domains. However, researchers have developed NK-specific intracellular signaling domains, such as DNAX-activating protein 10 (DAP10), DNAX-activating protein 12 (DAP12), and 2B4, to enhance their cytotoxicity and persistence (24).

Due to the dual cytotoxicity and activation mechanism of CAR-NK cell therapy, it shows greater potential in overcoming the heterogeneity of solid tumors over CAR-T cell therapy and is more effective in combination with radiotherapy for tumor treatment.

## 3 Challenges encountered by CAR-NK cell therapy in the treatment of solid tumors

### 3.1 Immune cell homing and transport

Immune cells are usually distributed in the peripheral blood and secondary lymphatic structures of the human body. When the body suffers from infectious or tumor-related diseases, a large number of inflammatory mediators like cytokines and histamines can be released to induce the migration of immune cells to the lesion site through the bloodstream. Then the immune cells adhere to the endothelial cells that line the blood vessels and permeate into the affected tissue via various cascades (25, 26). Nevertheless, solid tumors have abnormal vascular structure and function. Compared with normal blood vessels, their vasculature lacks hierarchical organization and shows a lower expression of adhesion molecules on the endothelium (27, 28), which hinders the migration of immune cells toward tumor tissues. The precise identification of tumors by CAR-NK cells and their successful migration to the tumor core through peripheral blood vessels are crucial prerequisites to ensure the efficacy of immune cell therapy.

### 3.2 Inhibitory effect of TME on CAR-NK cells

TME refers to the internal environment of tumor development, including tumor cells, immune cells, stromal cells, blood vessels, and extracellular matrix components (29). Abnormal growth of tumor blood vessels can lead to the hypoxic and acidic TME and make NK cells undergo several alterations, including decreased secretion of cytokines, downregulation of activating and death receptors (30), and decreased cytotoxicity. Conversely, lactate and low pH levels can inhibit the activity of NK cells and the IFNγ release (31). TME also releases various cytokines that suppress NK cell activity, such as prostaglandin E2 (PGE2), indoleamine 2,3-dioxygenase (IDO), extracellular adenosine, transforming growth factor β (TGF-β), and soluble MICA (sMICA) (32). Among these factors, TGF-β can significantly diminish the expression levels of activating receptors NKG2D and NKp30 on the NK cell surface (33). It can further disrupt IFN-γ generation and inhibit its differentiation into type 1 innate-like lymphoid cells (ILC1) (34, 35), thereby impairing the NK cell cytotoxicity. TME has diverse immunosuppressive cells, including regulatory T cells (Tregs), myeloid-derived suppressor cells (MDSCs), and tumor-associated macrophages (TAMs), all of which hinder NK cell activity. Tregs can inhibit NK cell activity by interacting with membrane-bound protein TGF-β and secreting TGF-β and IL-10 (36). Furthermore, Tregs compete with NK cells to bind IL-2 and suppress NK cell activation (37). MDSCs can impede NK cell-mediated ADCC via the release of nitric oxide (NO) (38). These cytokines and immunosuppressive cells do not act independently but function in a complex interaction.

## 4 Current status of CAR-NK cell therapy in the treatment of solid tumors

The currently available data on the efficacy of CAR-NK cell therapy primarily comes from hematologic malignancies. It has shown anti-tumor activity in various preclinical models of solid tumors (9, 39–42), including ovarian cancer, glioblastoma, pancreatic cancer, breast cancer, lung cancer, and others. Moreover, several CAR-NK cell therapies have been approved for treating certain cancers based on their promising performance in phase I/II clinical trials. Common target antigens include PSMA, ROBO1, and NKG2D, although their specific data has not been publicly disclosed. Currently, 13 clinical trials of CAR-NK cell therapy for solid tumors have been registered on ClinicalTrials.gov (**Table 2**).

**Table 2:** Registered clinical trials of CAR-NK cell therapy for treating solid tumors.

NCT number	Target	Cancer type	Trial phase	Trial status
NCT05776355	NKG2D	Ovarian Cancer	Phase I	Recruiting
NCT05507593	DLL3	SCLC, Extensive Stage	Phase I	Recruiting
NCT05410717	CLDN6	Stage IV Ovarian Cancer; Testis Cancer; Refractory, Recurrent Endometrial Cancer	Phase I/II	Recruiting
NCT05213195	NKG2D	Refractory, Metastatic Colorectal Cancer	Phase I	Recruiting
NCT05194709	5T4	Advanced Solid Tumors	Phase I	Recruiting
NCT04847466	PD-L1	Gastroesophageal Junction Cancer; Advanced HNSCC	Phase II	Recruiting
NCT05248048	NKG2D	Refractory, Metastatic Colorectal Cancer	Phase I/II	Unknown
NCT03940820	ROBO1	Solid Tumors	Phase I	Recruiting
NCT05922930	TROP2	Pancreatic Cancer; Ovarian Cancer; Adenocarcinoma	Phase I/II	Not yet recruiting
NCT03692663	PMSA	Metastatic Castration-Resistant Prostate Cancer	Phase I	Recruiting
NCT03692637	anti-Mesothelin	Epithelial Ovarian Cancer	Phase I	Unknown
NCT03415100	NKG2D	Solid Tumors	Phase I	Unknown

CAR-NK, chimeric antigen receptor-natural killer; SCLC, small-cell lung cancer; HNSCC, head and neck squamous cell carcinoma.

## 5 Principle of CAR-NK cell therapy combined with radiotherapy

Radiotherapy, as the cornerstone of cancer treatment, exerts its effects by releasing high-energy radiation that directly or indirectly damages cellular DNA (43–45). In addition to direct cytotoxicity towards tumor cells, it can induce a series of biological responses that have profound impacts on the immune system and TME.

**Figure 2:**
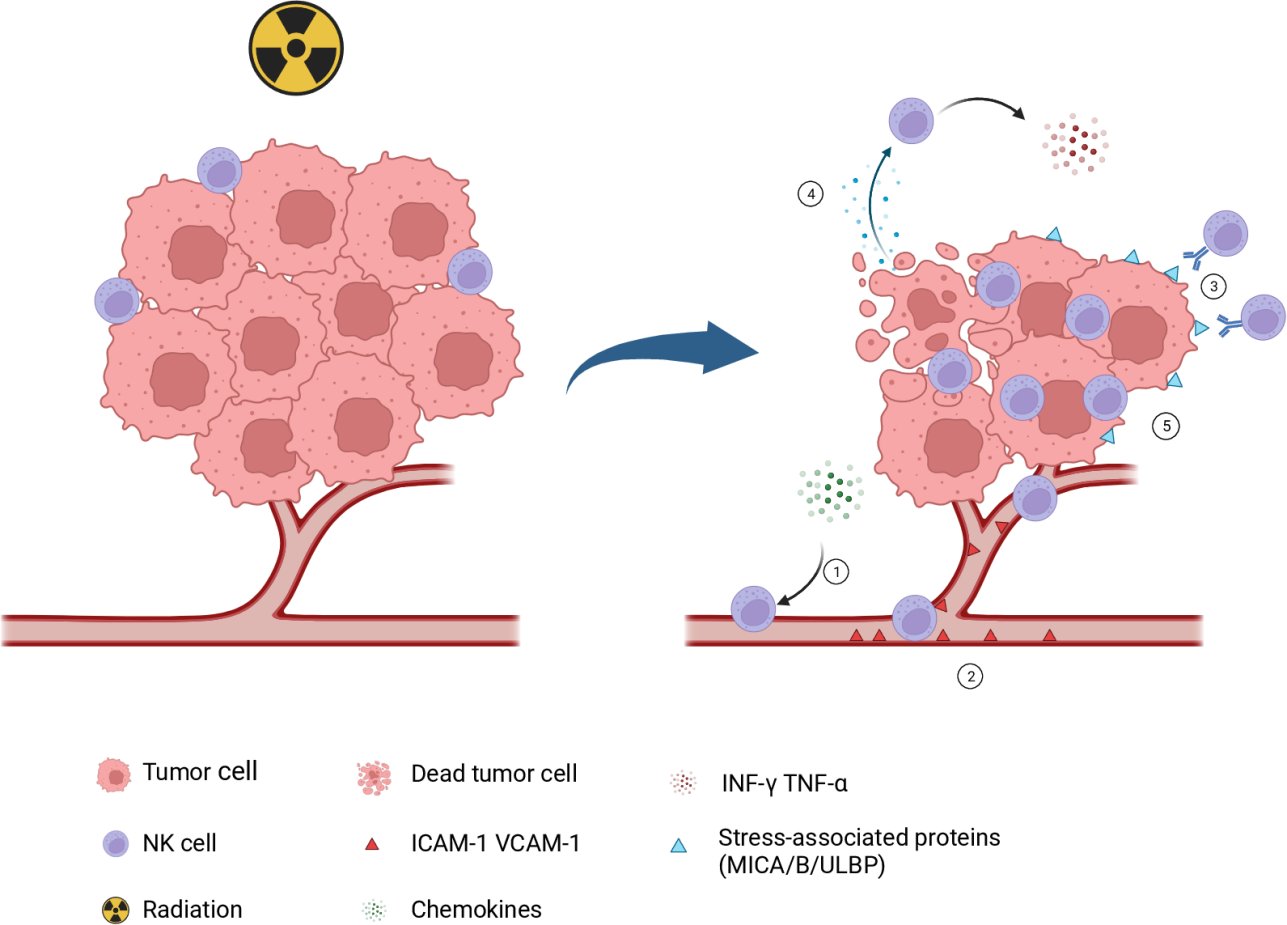
Role of NK cells in radiotherapy-induced antitumor immunity. 1.Following radiotherapy, the dsDNA of tumor cells is exposed to the cytoplasm to activate the cGAS/STING pathway, initiate type I interferon response, induce the secretion of some chemotactic factors, and recruit NK cells into the tumor microenvironment. 2. Radiotherapy can upregulate the expression of several adhesion molecules like ICAM-1 and VCAM-1, which increases the adhesion of NK cells to the endothelial surface. 3. After radiotherapy, tumor cells exhibit an upregulation in stress proteins MICA/B and ULBP1-6, which can effectively activate NK cells and initiate an immune response against the tumor. 4. Radiotherapy induces ICD in tumor cells, leads to the release of DAMPs, and activates NK cells. 5. Radiotherapy plays a pivotal role in decreasing tumor burden and creating a favorable environment for NK cell infiltration. The figure is created with BioRender.com NK, natural killer; cGAS, cyclic GMP-AMP synthase; STING, stimulator of interferon genes; ICAM-1, intercellular adhesion molecule 1; VCAM-1, vascular cell adhesion molecule 1; MIC, major histocompatibility complex class I chain-related protein. ULBP, UL16-binding protein; ICD, immunogenic cell death; DAMPs, damage-associated molecular patterns.

In 1953, Mole proposed the abscopal effect (46), which refers to the phenomenon where non-irradiated tumor lesions shrink in addition to the irradiated tumor lesions. This phenomenon is extremely rare and unpredictable. Subsequent researchers found that, in immune-deficient mouse tumor models, the radiation dose required to achieve tumor control was often higher than that in immune-competent mice (47), suggesting that the immune system contributes to enhancing the efficacy of radiotherapy. However, this did not receive much attention at the time. It was not until 2004 that Demaria et al. (48) confirmed that the abscopal effect induced by radiotherapy is mediated by the immune system. With the continuous progress of preclinical studies, more and more mechanisms have been discovered. Firstly, radiotherapy can induce immunogenic cell death (ICD) in tumor cells, leading to the release of damage-associated molecular patterns (DAMPs) (49–51). The primary components of DAMPs include: 1. The translocation of calreticulin from the endoplasmic reticulum to the cell membrane, which activates the antigen presentation function of dendritic cells (DCs), At the same time, calreticulin can also be recognized by the NKp46 receptor of NK cells, enhancing the cytotoxicity of NK cells (52–54). 2. The release of adenosine triphosphate (ATP), which recruits DC cells, macrophages, T cells, B cells, etc., to migrate to the injury site, releases inflammatory factors, and initiates adaptive immune responses (55). 3. The release of high mobility group protein B1 (HMGB1) promotes the maturation of dendritic cells, and then activates T cells (56). On the other hand, radiotherapy can alter the phenotype of tumor cells, leading to the upregulation of cell surface molecules and increasing the presentation of antigens, making the tumor cells more susceptible to immune cell attacks (57, 58). Lastly, radiotherapy can lead to the exposure of dsDNA, which is recognized and bound by cyclic GMP-AMP synthase (cGAS), and stimulates the production of many immune and inflammatory gene products, such as Type I interferons, through the cGAS/STING signaling pathway (59, 60). These pro-inflammatory factors recruit immune cells into the tumor, thereby remolding the tumor microenvironment and converting “cold” tumors with less immune cell infiltration into “hot” tumors with immune cell infiltration (**Figure 2**). Though preclinical studies illustrate the mechanisms of radiotherapy-induced abscopal effects, observation of these effects in clinical practice remains a rarity when radiotherapy is administered in isolation. This scarcity may be ascribable to the inability of radiotherapy alone to adequately surmount the cancer-induced immunosuppression.

With the emergence of immunotherapy, immune checkpoint inhibitors have been approved for clinical use, and an increasing number of abscopal effect cases have been reported. The first report came from a melanoma patient who experienced disease progression during a clinical trial of ipilimumab, but subsequently experienced tumor reduction outside of the irradiated field following radiotherapy (61). Meanwhile, a retrospective analysis of 24 cases confirmed that abscopal effects are more common in patients receiving combined radiotherapy and immunotherapy (62). The impact of radiotherapy on the immune system has regained attention. Researchers have begun to explore combinations of radiotherapy with various immunotherapies, including immune checkpoint inhibitors, adoptive cell therapy, cytokine therapy, etc. Currently, there are over hundreds of ongoing clinical trials combining radiotherapy with different immunotherapies.

It is generally believed that radiotherapy-induced antitumor immune responses are primarily mediated by T cells. A large body of research has also confirmed the crucial role of T cells in radiotherapy-induced immune responses (48, 63, 64). However, with the development of immunology, researchers have begun to pay attention to the role of NK cells in this process. We will subsequently introduce the role of NK cells in radiotherapy-induced antitumor immunity, as well as the potential mechanism of combining CAR-NK with radiotherapy.

### 5.1 Radiotherapy enhancing NK cell homing and transport

Compared with normal blood vessels, tumor blood vessels exhibit immature morphology, irregular distribution, and increased permeability. The effects of radiation therapy on tumor vasculature vary greatly and depend on several factors like radiation dose, fractionation schedule, tumor type, and tumor location. Typically, a single high dose exceeding 10 Gy can cause endothelial cell death, acute vascular injury, reduced blood perfusion, and cell hypoxia within hours (65). However, moderate to low doses can stimulate vascular regeneration and induce vascular normalization. Studies have reported that a radiation dose ranging from 5-10 Gy can transiently restore normal vascular function by inducing NO release and lead to tumor reoxygenation, improved blood flow, and increased tumor perfusion (66). Notably, Ganss et al. demonstrated that irradiation of the RIP1-Tag5 mice of pancreatic islet tumor with a dose of 10 Gy could normalize tumor blood vessels and enhance lymphocyte infiltration into the tumor (67). Similarly, Potiron et al. found that a fractionated radiation regimen (2 Gy per fraction, 5 fractions per week, a total dose of 20 Gy in 10 fractions) could improve tumor vascular maturity and perfusion and reduce cell hypoxia in a prostate cancer xenograft model, and it did not alter the tumor vascular morphology or density (68). Subsequent experiments have confirmed that low-dose radiation therapy can normalize tumor vasculature and enhance drug distribution within the tumor (69). Additionally, radiation therapy can increase the expression of adhesion molecules like intercellular adhesion molecule-1 (ICAM-1) and vascular cell adhesion molecule-1 (VCAM-1) on tumor endothelial cells (70, 71). These adhesion molecules facilitate the adherence of NK cells to the endothelial cells and promote extravasation into the tumor. Based on these findings, the combination of radiation therapy with CAR-NK cell therapy may enhance CAR-NK cell homing and delivery, and the vascular normalization induced by radiation therapy may reverse the inhibitory effect of hypoxia on CAR-NK cells.

### 5.2 Radiotherapy increasing NK cell infiltration and recognition

The infiltration degree of NK cells in tumor tissues is often positively correlated with the patient’s prognosis (72–74). Radiotherapy plays a crucial role in NK cell migration and infiltration. A study on single-cell sequencing of the paired cervical cancer samples before and after radiochemotherapy revealed a substantially increased number of CD16-NK cells in tumor tissues following treatment. Analysis of the transcriptome data on infiltrating NK cells after radiochemotherapy demonstrated an increased expression of genes associated with leukocyte migration and cytotoxicity (75). Similar results were obtained in endometrial cancer and colorectal cancer, where radiotherapy increased NK cell infiltration (76, 77). The influence of radiotherapy on NK cell migration may be attributed to the regulation of several chemokines. NK cells have many chemokine receptor proteins on their surface, such as CXCR1, CXCR2, CXCR3, CXCR6, and CCR5. These receptors facilitate the tumor cells’ perception of signal gradients of specific chemokines and guide their migration process. Moreover, radiotherapy can induce DNA damage, activate the cGAS/STING signaling pathway, trigger a type I interferon response, and promote the production of some cytokines like CXCL9, CXCL10, and CXCL11 (78–80). These cytokines bind to receptors on NK cells and enhance immune cell migration and infiltration into the tumor site (81).In addition, radiotherapy-induced production of CXCL8 with CXCL16 has been reported to be associated with NK cell migration and infiltration (82, 83).

Radiotherapy can induce the secretion of multiple chemokines, upregulate the expression of tumor-specific surface antigens, and enhance the recognition and activation of NK cells. Research has demonstrated that radiotherapy at a dose of 20 Gy can increase the expression of MICA/B and ULBP1/2 on the surface of tumor cells, and, co-culture with these tumor cells can enhance the cytotoxicity of NK cells (84). Another study analyzed the paired samples from sarcoma patients before and after radiotherapy; the post-radiotherapy tumor cells exhibited stem cell characteristics and an upregulated expression of MICA/B, making them more susceptible to NK cell-mediated cytotoxicity (85). Moreover, radiotherapy upregulates the expression of ICAM1 on cell surface, promotes the adhesion between NK and tumor cells, and enhances NK cell-mediated toxicity (86).The radiotherapy combined with adoptive NK cell therapy could significantly prolong overall survival and suppress lymph node metastasis and distant metastasis in a mouse model of triple-negative breast cancer. Besides, radiotherapy and adoptive NK cell therapy showed favorable results in canine sarcoma models by enhancing NK cell homing and NK cell-mediated cytotoxicity (87, 88).

In conclusion, these preclinical studies show that radiotherapy can enhance the infiltration ability and tumor recognition ability of NK cells in adoptive therapy (**Table 3**).

**Table 3:** Preclinical evidence for radiotherapy combined with NK cells.

Tumor model	Radiation Dose	Mechanism	Reference
Breast Cancer	12 Gy×1	Radiation therapy enhanced the infiltration of NK cells into tumors and improved the tumor control rate.	Kim et al. (87)
Pancreatic Cancer	20 Gy×1	Radiation therapy induced CXCL8 secretion and promoted the infiltration of NK cells into tumors.	Walle et al. (82)
Breast Cancer Cell Lines; Colorectal Cancer Cell Lines	20 Gy×1	Radiotherapy increased ICAM-1 expression and enhanced NK cell cytotoxicity.	Jeong et al. (86)
Breast Cancer Cell Lines	20 Gy×1	Radiation therapy enhanced CXCL16 secretion and induced the migration of NK cells toward tumor cells.	Yoon et al. (83)
Melanoma; Lung Cancer; Cervical Cancer Cell Lines	20 Gy×1	Radiotherapy increased the expression of the NKG2D ligand on the surface of tumor cells and the cytotoxicity of NK cells.	Kim et al. (84)
Sarcomas	9 Gy×1	Radiotherapy increased the cytotoxicity and homing of NK cells.	Canter et al. (88)
Nasopharyngeal Carcinoma	2 Gy×1	RT combined with immunotherapy increased the cytotoxicity of NK cells.	Laurent et al. (89)

NK, natural killer; RT, radiotherapy; CXCL8, C-X-C motif chemokine ligand 8; CXCL16, C-X-C motif chemokine ligand 16; ICAM-1, intercellular adhesion molecule 1; NKG2D, natural killer group 2 member D.

### 5.3 Radiation therapy reducing tumor burden and heterogeneity

Solid tumors often display significant heterogeneity, which can lead to antigen escape during CAR-NK cell therapy. Radiotherapy can effectively kill a substantial number of tumor cells and reduce tumor heterogeneity. Furthermore, it can decrease tumor burden and create a more favorable environment for immune cell infiltration. Previous literature has reported that patients with lower tumor burdens have better response rates and outcomes to CAR-T cell therapy (90).

## 6 Challenges faced by the combination therapy

### 6.1 Radiotherapy-mediated immunosuppression

Although radiotherapy and adoptive NK cell therapy have shown promising synergistic effects in clinical or preclinical experiments, the immunosuppressive effects brought by radiotherapy cannot be ignored. Radiotherapy can directly exert cytotoxic effects on immune cells. Several studies have reported reduced number and activity of NK cells in patients following radiotherapy (91, 92), indicating that NK cell therapy should be administered after radiotherapy and highlighting the significance of its initiation timing in combination treatment. Moreover, radiotherapy can recruit immunosuppressive cells from TME, such as Tregs and MDSCs. Numerous studies have shown a significantly increased number of intratumoral Tregs after radiotherapy (93–95), while Tregs can exert strong inhibitory effects on NK cells (96). The precise mechanism of radiotherapy-induced expansion of Tregs has not yet been fully elucidated. Recent research has revealed that radiotherapy at a dose of 10 Gy prioritizes stimulating proliferation of pre-existing intratumoral Tregs, rather than recruiting peripheral blood Tregs into the tumor, and this study further revealed that TGFβ and IL33 signaling pathways were irrelevant to Treg expansion (93). Furthermore, a study on a mouse model of head and neck squamous cell carcinoma demonstrated that high-dose radiotherapy induced tumor cells to secrete the chemokine CCL20, which could enhance Tregs to infiltrate into the tumor tissue via the CCR6-CCL20 axis and exert their immunosuppressive effects (97). These studies indicate that radiotherapy-induced infiltration of Tregs into tumors may involve various factors, including tumor type, radiation dose, and fractionation schedule. The effectiveness of radiotherapy combined with targeted inhibitors against Tregs has been confirmed in some preclinical studies (98). Radiation therapy can induce the release of chemokine CCL2 to recruit MDSCs, while CCL2 can activate the CCL2/CCR2 and CCL2/CCR5 signaling pathways, leading to MDSCs infiltration into the TME and immune suppression (99–101). Moreover, radiation therapy can modulate the expression of tumor-associated antigens like MHC class I molecules (58, 102), decrease the NK cell’s ability to recognize the tumor. Therefore, it is crucial to address the immunosuppressive effects induced by radiation therapy and maximize the synergistic effects of radiotherapy and CAR-NK cell therapy. Further experiments are warranted to gain deeper insights into the complex interaction between radiation therapy and immunotherapy.

### 6.2 Optimal radiotherapy dose and fractionation scheme

There is a lack of clinical and preclinical data supporting the therapeutic efficacy of the combination of radiotherapy with CAR-NK cell therapy, and the optimal dosage and schedule of radiotherapy remain unclear. A critical issue is whether higher ablative doses or lower doses of radiotherapy can yield better outcomes in combination therapy. Furthermore, different cancers may exhibit distinct responses to the combination therapy. The higher ablative doses of radiotherapy may promote ICD, release DAMPs, and activate immune cells (103). Nevertheless, its low dose may reverse tumor immune desertification and increase the infiltration of immune cells into tumors (104).

Numerous studies have shown an increased infiltration of NK cells into tumor tissues after administering standard radiation doses of 50-60 Gy (75, 77, 105). A preclinical investigation aimed to explore the impact of dose per fraction (DPF) and cumulative dose on the immunomodulatory capability of radiotherapy. Utilizing an AT3-OVA mouse model, it was observed that lower DPFs (3x4 Gy, 9x4 Gy, 3x8 Gy) could significantly stimulate CD8+ T cell-mediated antitumor activity compared with a single administration of a high dose fraction (1x12 Gy, 1x20 Gy). Conversely, the activation of NK cell-mediated antitumor function was found to be dependent on the cumulative dose rather than the DPF, and its remarkable enhancement was observed after surpassing a biologically effective dose of 36 Gy. These findings strongly suggest the application of conventional radiotherapy doses in combination with CAR-NK cell therapy (106). A dose threshold exceeding 7.5 Gy in the single high-dose radiation therapy has been shown to increase Treg infiltration and lead to immune suppression. Therefore, it is advised to set the radiotherapy dose below this threshold to optimize treatment outcomes and minimize immunosuppressive effects (107). Further research is required to determine the most effective dosage and fractionation schedule of radiotherapy in combination with CAR-NK cell therapy. The existing evidence suggests that ablative high-dose and low-dose radiotherapy have advantages in immunogenicity and immune activation, but their efficacies may vary depending on the specific cancer model.

### 6.3 Therapeutic sequence

Radiation is commonly regarded as detrimental to NK cells, leading to apoptosis and dysfunction of these cells (108). Therefore, it is recommended that radiotherapy be administered prior to CAR-NK cell infusion to prevent direct damage to the NK cells. In recent years, advancements in radiotherapy technology have allowed for greater precision in dose delivery, minimizing damage to surrounding blood vessels, lymph nodes, and normal tissues, and subsequently reducing the impact on immune cells (109). However, the timing of radiotherapy intervention within the context of combination therapy remains a critical factor. Min Zhou et al. (14) conducted a study exploring the optimal timing of radiotherapy intervention in a breast cancer mouse model treated with a CAR-T cell therapy and radiotherapy combination. Their results showed no statistically significant differences in tumor volume control and survival of mice when administering radiotherapy before or after infusion. This research is the sole study investigating the treatment sequence of adoptive immunotherapy in combination with radiotherapy.

**Figure 3:**
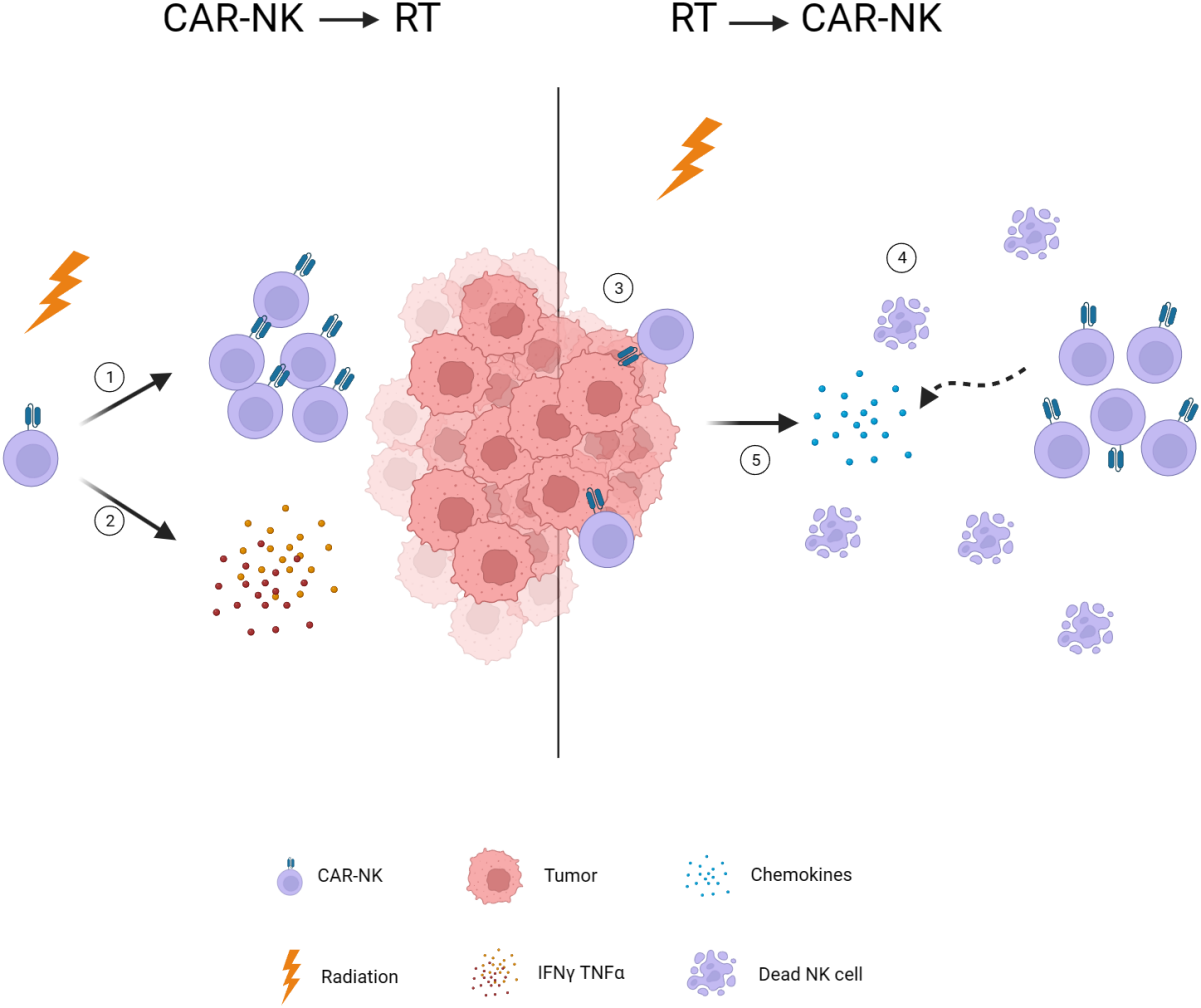
Timing of radiotherapy intervention. 1. Low-dose radiotherapy induces NK cell expansion. 2. Low-dose radiotherapy stimulates NK cells to secrete INF-γ and TNF-α. 3. Radiotherapy reduces tumor burden and facilitates CAR-NK infiltration. 4. Radiotherapy-induced lymphopenia provides space for CAR-NK expansion. 5. Radiotherapy induces chemokine release and attracts CAR-NK to the tumor. CAR, chimeric antigen receptor; NK, natural killer; TNF-α, tumor necrosis factor alpha; IFN-γ, interferon-gamma.

According to previous studies, the post-radiotherapy administration of CAR-NK cells appears to be an effective approach for several reasons. Firstly, the lymphopenia resulting from radiotherapy may cultivate an optimal milieu for the proliferation of the introduced CAR-NK cells. similar to the pretreatment before CAR-T infusion (110, 111). Furthermore, lasting chemokine release is precipitated by radiotherapy enhancing the chemotaxis of these immune cell (82, 83). Also, radiotherapy mediates a significant reduction in tumoral burden, thereby improving the potential for efficient tumoral infiltration by the subsequent CAR-NK cell administration. Overall, these findings are more supportive of using CAR-NK after radiotherapy. Notably, research has indicated that a single low dose of radiation (<2Gy) can enhance the expansion and cytokine secretion function of NK cells *in vitro* (112, 113). Simultaneously, low-dose radiation therapy in a mouse model has been shown to increase the cytotoxic function of NK cells, consequently inhibiting the growth of experimental tumor (114, 115). Due to the limited duration of CAR-NK cells in the body (8), multiple injections are required to maintain therapeutic efficacy. The application of low-dose radiotherapy following CAR-NK cell infusion may represent a potential strategy for bolstering the functionality and durability of CAR-NK cells. Additional research and thorough investigation are crucial to validate the effectiveness of this potential therapeutic strategy (**Figure 3**).

## 7 Discussion

Based on the aforementioned preclinical research findings, a theoretical basis is provided for the combined therapy of NK cells and radiotherapy. A phase II randomized controlled trial reported favorable results from the use of autologous NK cell adoptive therapy following chemo-radiotherapy in non-small cell lung cancer (116). At present, multiple clinical trials using non-engineered NK cells combined with therapies for solid tumors, including chemo-radiotherapy, immune checkpoint inhibitors, and targeted therapies, are under way. Furthermore, an increasing number of researchers are beginning to use engineered NK cells for combination therapy. Apart from endowing NK cells with innate cytotoxicity, engineered NK cells have been given additional functionalities, potentially increasing their effectiveness in combined therapy. Though clinical data for CAR-NK therapy of solid tumors have yet to be published, and clinical trials combining it with radiotherapy have not started, we hold an optimistic view of the potential of this combination therapy. Firstly, in the case of solid tumors such as glioblastoma, pancreas and ovarian cancer, they are usually diagnosed at a late stage and generally show resistance to existing treatments, with almost no current targeted treatment options. CAR-NK cells may demonstrate a synergistic effect with standard treatments (chemotherapy, radiotherapy, targeted therapies), offering a possible treatment option for late-stage patients. The aim is to improve the targeting, endurance, chemotaxis, and safety of CAR-NK cells. current development strategies for CAR-NK therapy in solid tumors focus on identifying reliable new targets and constructing dual-targeted or multi-targeted CAR structures to enhance targeting, durability, chemotaxis, and safety. The increased exposure of neoantigens and expression of chemokines in tumor cells induced by radiotherapy may offer new avenues for CAR-NK therapy development, such as CARs targeting NKG2DL or overexpressing chemokine receptors (CXCR1, CXCR2, and CXCR4) through CAR design (117–120).

## 8 Conclusion

In summary, this article reviews the role and latest advancements of NK cells in radiotherapy-induced anti-tumor immunity. On the basis of this evidence, we propose that a combination of CAR-NK cell therapy and radiotherapy may be a method to overcome solid tumors. This hypothesis could provide treatment options for patients with advanced solid tumors and also offer strategies for the development of CAR-NK. However, to determine the optimal radiation dosage, fractionation scheme, and order of administration, a substantial amount of preclinical and clinical experiments is still necessary. Moreover, identifying specific biomarkers is crucial for selecting appropriate patients and minimizing potential treatment-related toxicity to the maximum extent.

## Author contributions

JH: Writing – original draft. YY: Investigation, Writing – review & editing. JZ: Writing – review & editing. ZW: Writing – review & editing. HL: Writing – review & editing. LX: Conceptualization, Writing – review & editing.
